# Supplementary material for: Identification of photoperiod-induced specific miRNAs in the adrenal glands of Sunite sheep (Ovis aries)
Source: Front Vet Sci. 2022 Jul 22;9:888207. doi: 10.3389/fvets.2022.888207 (PMC9354845; doi:10.3389/fvets.2022.888207)
Supplement: Supplementary file 1 [file Data_Sheet_1.ZIP › Supplementary file/miRDeep2 file.zip!/miRDeep2 file/Know_Structure/SPLP42A3/oar-mir-99a.pdf]

miRBase precursor : oar-mir-99a
Total read count : 1389680
oar-miR-99a read count : 1389422
remaining reads : 258

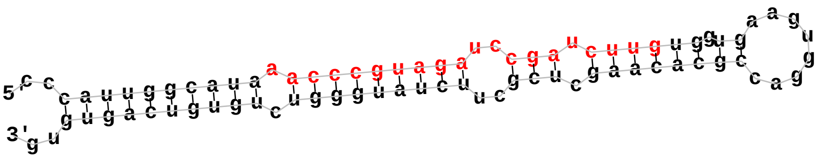

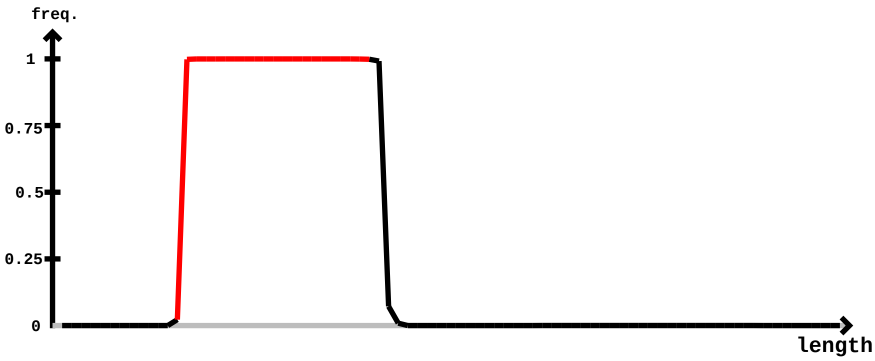

oar-miR-99a

5'- cccauuggcauaaacccguagauccgaucuuguggugaaguggaccgcacaagcucgcuucuaugggucugugucagugug -3'	exp		
..((((((((((.(((((((((..(((.((((((.((........)))))))).))).)))))))))).))))))))))..	reads	mm	sample
..........uaaacccguagauccgaucuugu..	7	0	seq
..........uaaacccguagauccgaucuugug...	1	0	seq
...........aaacccguagauccgau...	1	0	seq
...........aaacccguagauccgaucu...	10	0	seq
...........aaacccguagauccgaucuu..	19	0	seq
...........aaacccguagauccgaucuug...	82	0	seq
...........aaacccguagauccgaucuugu..	30614	0	seq
...........aaacccguagauccgaAcuugu..	6	1	seq
...........aaacccguagauccgaucuugug...	191	0	seq
...........aaacccguagauccgaucuugugg..	7	0	seq
............aacccguagauccgauc..	58	0	seq
............aacccguagauccgaucu...	423	0	seq
............aacccguagauccgaAcu...	31	1	seq
............aacccguagauccgaucuu..	1208	0	seq
............aacccguagauccgaAcuu..	84	1	seq
............aacccguagauccgaAcuug...	690	1	seq
............aacccguagauccgaucuug...	8466	0	seq
............aacccguagauccgaucuugu..	1236443	0	seq
............aacccguagauccgaAcuugu..	9419	1	seq
............aacccguagauccgaAcuugug...	15020	1	seq
............aacccguagauccgaucuugug...	71968	0	seq
............aacccguagauccgaAcuugugg..	172	1	seq
............aacccguagauccgaucuugugg..	12260	0	seq
............aacccguagauccgaucuuguggu...	27	0	seq
............aacccguagauccgaAcuuguggu...	3	1	seq
.............acccguagauccgaucuu..	1	0	seq
.............acccguagauccgaAcuug...	9	1	seq
.............acccguagauccgaucuug...	11	0	seq
.............acccguagauccgaucuugu..	1436	0	seq
.............acccguagauccgaAcuugu..	124	1	seq
.............acccguagauccgaucuugug...	99	0	seq
.............acccguagauccgaAcuugug...	280	1	seq
.............acccguagauccgaAcuugugg..	7	1	seq
.............acccguagauccgaucuugugg..	17	0	seq
..............cccguagauccgaucuugu..	38	0	seq

oar-miR-99a

```
cccauuggcauaaacccguagauccgaucuuguggugaaguggaccgcacaagcucgcuucuaugggucugugucagugug
..............cccguagauccgaAcuugug...............................................    1     1     seq
..............cccguagauccgaucuugugg..............................................    1     0     seq
...............ccguagauccgaucuugu................................................    85    0     seq
...............ccguagauccgaucuugug...............................................    5     0     seq
...............ccguagauccgaAcuugug...............................................    3     1     seq
...............ccguagauccgaucuugugg..............................................    2     0     seq
................cguagauccgaucuugu................................................    83    0     seq
................cguagauccgaucuugug...............................................    7     0     seq
................cguagauccgaucuugugg..............................................    3     0     seq
.................................................caagcucgcuucuaugggucu...........    1     0     seq
.................................................caagcucgcuucuaugggucug..........    15    0     seq
.................................................caagcucgcuucuaugggucugu.........    224   0     seq
.................................................caagcucgcuucuaugggucugug........    1     0     seq
..................................................aagcucgcuucuaugggucugu.........    16    0     seq
...................................................agcucgcuucuaugggucugu.........    1     0     seq
```
